# The spectrum of low molecular weight alpha-amylase/protease inhibitor genes expressed in the US bread wheat cultivar Butte 86

**DOI:** 10.1186/1756-0500-4-242

**Published:** 2011-07-20

**Authors:** Susan B Altenbach, William H Vensel, Frances M Dupont

**Affiliations:** 1 USDA-ARS Western Regional Research Center, 800 Buchanan Street, Albany, CA 94710, USA

**Keywords:** allergens, expressed sequence tags, plant defense proteins, tandem mass spectrometry

## Abstract

**Background:**

Wheat grains accumulate a variety of low molecular weight proteins that are inhibitors of alpha-amylases and proteases and play an important protective role in the grain. These proteins have more balanced amino acid compositions than the major wheat gluten proteins and contribute important reserves for both seedling growth and human nutrition. The alpha-amylase/protease inhibitors also are of interest because they cause IgE-mediated occupational and food allergies and thereby impact human health.

**Results:**

The complement of genes encoding alpha-amylase/protease inhibitors expressed in the US bread wheat Butte 86 was characterized by analysis of expressed sequence tags (ESTs). Coding sequences for 19 distinct proteins were identified. These included two monomeric (WMAI), four dimeric (WDAI), and six tetrameric (WTAI) inhibitors of exogenous alpha-amylases, two inhibitors of endogenous alpha-amylases (WASI), four putative trypsin inhibitors (CMx and WTI), and one putative chymotrypsin inhibitor (WCI). A number of the encoded proteins were identical or very similar to proteins in the NCBI database. Sequences not reported previously included variants of WTAI-CM3, three CMx inhibitors and WTI. Within the WDAI group, two different genes encoded the same mature protein. Based on numbers of ESTs, transcripts for WTAI-CM3 Bu-1, WMAI Bu-1 and WTAI-CM16 Bu-1 were most abundant in Butte 86 developing grain. Coding sequences for 16 of the inhibitors were unequivocally associated with specific proteins identified by tandem mass spectrometry (MS/MS) in a previous proteomic analysis of milled white flour from Butte 86. Proteins corresponding to WDAI Bu-1/Bu-2, WMAI Bu-1 and the WTAI subunits CM2 Bu-1, CM3 Bu-1 and CM16 Bu-1 were accumulated to the highest levels in flour.

**Conclusions:**

Information on the spectrum of alpha-amylase/protease inhibitor genes and proteins expressed in a single wheat cultivar is central to understanding the importance of these proteins in both plant defense mechanisms and human allergies and facilitates both breeding and biotechnology approaches for manipulating the composition of these proteins in plants.

## Background

Wheat kernels accumulate a variety of low molecular weight proteins that inhibit amylases and/or proteases from different sources. The first report of alpha-amylase inhibition by purified wheat proteins was by Silano et al. [1]. Three groups of alpha-amylase inhibitors have been described that are active against insect, mite and mammalian alpha-amylases, but not against cereal enzymes (reviewed by [2]). These include the 12 kDa monomeric inhibitors (WMAI), often referred to as 0.28 proteins, encoded by genes on the short arms of the group 6 chromosomes; proteins that form the 24 kDa homodimeric inhibitors (WDAI), sometimes referred to as the 0.19 and 0.53 proteins, encoded by genes on the short arms of the group 3 chromosomes; and proteins that make up the 60 kDa heterotetrameric inhibitors (WTAI). The tetrameric inhibitors are often referred to as CM proteins because of their solubility in chloroform/methanol. They generally are composed of one copy of either CM1 or CM2, encoded by genes on chromosomes 7D or 7B, plus one copy of either CM16 or CM17, encoded by genes on chromosomes 4B or 4D, plus two copies of CM3, also encoded on chromosomes 4B or 4D. The inhibitory activity of the WTAI is dependent on the combination of subunits [3]. A number of other proteins share structural similarities to the alpha-amylase inhibitors but are active against specific proteases. Thus far, these proteins have been characterized in barley and other cereals, but not in wheat. However, Sanchez de la Hoz et al. [4] isolated several wheat cDNAs that encoded proteins similar to the barley trypsin inhibitor BTI-CMe. The putative wheat trypsin inhibitors are referred to as CMx proteins and are encoded by genes on the group 4 chromosomes. Another putative protease inhibitor, referred to as WCI, may inhibit chymotrypsin, but information about this protein is limited to what is contained in a single cDNA entry in NCBI [GenBank: AJ422078]. All of the mature alpha-amylase/protease inhibitor proteins contain 10 cysteine residues that form five disulfide bonds. Proteins in another class, referred to as WASI, inhibit endogenous alpha-amylases. These proteins are bi-functional and can also inhibit subtilisin. The WASI proteins differ from the exogenous alpha-amylase and protease inhibitors in that they contain only 4 cysteine residues. Several recent proteomic studies in the bread wheat cv. Butte 86 [5] and the durum wheat cv. Svevo [6] noted that the levels of WASI increased in developing grain subjected to high temperatures, suggesting that this protein may be involved in the response of the grain to abiotic stress. Yang et al. [7] also reported changes in the levels of WMAI, WDAI and the WTAI subunits CM1, CM3 and CM17 in grain from the bread wheat cv. Vinjett subjected to different combinations of temperature and water stress.

In addition to playing a protective role, the alpha-amylase/protease inhibitors are accumulated to sufficiently high levels in the grain to function as storage proteins. In contrast to the major gluten proteins that contain large percentages of glutamine and proline, the alpha-amylase/protease inhibitors have more balanced amino acid compositions. As a result, these proteins compensate in part for deficiencies in essential amino acids in the gluten proteins and contribute important reserves for both seedling growth and human nutrition.

The alpha-amylase/protease inhibitors are also of interest because many are involved in wheat allergies (reviewed by [8]). The WMAI, WDAI and WTAI subunits have been shown to be a major cause of baker's asthma, an important occupational allergy that affects millers and bakers. A glycosylated form of the WTAI subunit CM16 is likely to be the most allergenic of the amylase/protease inhibitors since it exhibited the strongest binding to IgE from patients with baker's asthma in immunoblots [9]. Additionally, the trypsin inhibitor from barley that is similar to the wheat CMx inhibitors has been shown to be an allergen [10]. The WDAI and WTAI also have been implicated in IgE-mediated food allergies in a number of studies that combined immunoblotting with mass spectrometry [11,12].

A number of recent studies have addressed the diversity of sequences for the WMAI and WDAI in cultivated wheat and wheat relatives [13-15]. This information is valuable since WDAI proteins with 98% sequence identity can exhibit different specificities against alpha amylases from insect and mammalian sources [16]. Thus far, there is little information about the diversity of proteins within the WTAI because the various subunits of the tetrameric inhibitor have been sequenced from only a few wheat varieties. Additionally, few studies have examined the spectrum of amylase/protease inhibitors accumulated in single cultivars.

A recent study by Dupont et al. [17] used 2-dimensional gel electrophoresis (2-DE) combined with tandem mass spectrometry (MS/MS) to identify proteins in a total milled flour sample from the US bread wheat Butte 86. Proteins in 21 of the 233 abundant protein spots analyzed in this study were identified as alpha-amylase/protease inhibitors. These corresponded to 16 different protein sequences, but none were from Butte 86 because sequences of alpha-amylase/protease inhibitors from Butte 86 were not included in the database used for analysis of MS/MS data. Nine inhibitors matched sequences in NCBI. Six of these were deduced from genomic DNA or cDNA sequences. The other three were protein sequences so the corresponding gene sequences could only be inferred from tblastn searches. Seven other inhibitors matched proteins deduced from consensus sequences of contigs from large contig databases, six from DFCI Wheat Gene Index Release 11.0 [18] and one from HarvEST 1.14 [19]. Contig databases contain a wealth of information. However, contig consensus sequences are tentative and change as databases are updated with new ESTs and assemblies. To link individual protein spots from the Dupont et al. [17] study to the precise DNA sequences for alpha-amylase/protease inhibitors from Butte 86, we have now examined the complement of genes expressed in Butte 86 by assembling ESTs from this cultivar. We describe differences between the Butte 86 sequences and previously published sequences and relate peptides obtained by MS/MS in the previous proteomic analysis of Butte 86 flour [17] to proteins encoded by the Butte 86 sequences.

## Results and Discussion

Ninety-seven ESTs from the US hard red spring wheat Butte 86 were assembled into 19 contigs encoding low molecular weight alpha-amylase/protease inhibitors (Table 1, Additional files 1, 2). Two additional ESTs were excluded from the study because they did not align with other sequences and were of poor quality. Three inhibitors were represented by single ESTs, while contigs for other inhibitors were comprised of between two and 20 ESTs. Five of the contigs contained coding regions that were perfect matches with *T. aestivum *sequences in NCBI nr and three were perfect matches with sequences from *T. turgidum*. Sequences of proteins deduced from Butte 86 contig consensus sequences are shown in Additional file 3. Mature proteins encoded by the contigs ranged from 12,962 to 19,690 in MW. All but four of the proteins were between ~13,000 and 14,000 MW. Isoelectric points of the proteins ranged from 4.87 to 8.08, with 11 clustered between 6.14 and 6.77 (Table 1).

**Table 1:** Identification of Butte 86 contigs for alpha-amylase/protease inhibitors, similarity to sequences in NCBI and characteristics of encoded proteins

Butte 86 Contig Name	# ESTs	Representative EST	Coding region bp	Closest match from *T. aestivum *in NCBI nr^1^	Identity	Cultivar	MW^2^	#aa^2^	pI^2^
WMAI Bu-1	11	[Genbank:BQ804583]	456	[Genbank:AK336166]	456/456	Chinese Spring	13155	121	6.19
WMAI Bu-2	3	[Genbank:BQ805680]	456	[Genbank:AK336166]^3^	440/456	Chinese Spring	13056	121	5.37
WDAI Bu-1	5	[Genbank:BQ805792]	465	[Genbank:AK330823]^4^	438/465	Chinese Spring	13337	124	6.66
WDAI Bu-2	2	[Genbank:BQ805854]	465	[Genbank:DQ019829]	426/426	Chinese Spring	13337	124	6.66
WDAI Bu-3	2	[Genbank:BQ805829]	465	[Genbank:AK330823]	464/465	Chinese Spring	13181	124	6.49
WDAI Bu-4	1	[Genbank:BQ806690]	459	[Genbank:DQ019827]^5^	425/426	Chinese Spring	13191	124	5.23
WTAI-CM1 Bu-1	8	[Genbank:BQ804568]	438	[Genbank:X17575]	437/438	Chinese Spring	13095	120	6.72
WTAI-CM2 Bu-1	8	[Genbank:BQ804416]	438	[Genbank:X17575]^6^	415/440	Chinese Spring	13034	120	6.23
WTAI-CM3 Bu-1	20	[Genbank:BQ804159]	507	[Genbank:AK330649]	507/507	Chinese Spring	15832	143	6.66
WTAI-CM3 Bu-2	7	[Genbank:BQ804161]	507	[Genbank:AK330649]	484/507	Chinese Spring	15916	143	6.66
WTAI-CM16 Bu-1	10	[Genbank:BQ804374]	432	[Genbank:X17573]	432/432	Chinese Spring	13437	119	5.02
WTAI-CM17 Bu-1	5	[Genbank:BQ804549]	432	[Genbank:X59791]	431/431	Timaglen	13502	120	4.87
WASI Bu-1	2	[Genbank:BQ806745]	543^7^	[Genbank:AK334580]	529/546	Chinese Spring	19633	180	6.77
WASI Bu-2	3	[Genbank:BQ806454]	612^8^	[Genbank:AK334580]	611/612	Chinese Spring	19690	181	6.77
CMx Bu-1	2	[Genbank:BQ805931]	438	[Genbank:X75608]	397/425	Chinese Spring	14009	122	6.14
CMx Bu-2	4	[Genbank:BQ806494]	441	[Genbank:X75608]	426/441	Chinese Spring	14027	122	8.08
CMx Bu-3	1	[Genbank:BQ804611]	435	[Genbank:X75608]	395/422	Chinese Spring	13891	121	8.02
WTI Bu-1	1	[Genbank:BQ806495]	417^9^	nd^10^	na^11^	na^11^	13290	122	5.47
WCI Bu-1	2	[Genbank:BQ805289]	432	[Genbank:AJ422078]	347/348	San Pastore	12962	119	7.42

^1 ^Determined by BLASTn of NCBI nr database limited to *T. aestivum *using coding region only, database last searched on 4/1/11.

^2 ^Mature protein encoded by contig after signal peptide cleavage at site predicted by Signal P [20]. Assumes additional N-terminal processing for WMAI and WDAI.

^3 ^Coding region is a perfect match with [Genbank:FJ874627] from *T. turgidum *subsp. dicoccoides isolate 33-13A that was added to NCBI after database used for MS/MS analysis in Dupont et al. [17] was created.

^4 ^425/426 bp of coding region matches [Genbank:FJ897099] from *Aegilops tauschii *PI499264-2(D) that was added to NCBI after database used for MS/MS analysis in Dupont et al. [17] was created.

^5 ^Coding region is a perfect match with [Genbank:FJ897030] from *T. turgidum *subsp. dicoccoides isolate TD30-49-18 that was added to NCBI after database used for MS/MS analysis in Dupont et al. [17] was created.

^6 ^Coding region is a perfect match with [Genbank:X55454] from *T. turgidum *subsp. durum cv Desp. GATH.

^7 ^Does not include coding sequences for signal peptide.

^8 ^Assumes that C at position 537 of BQ806454 is deleted.

^9 ^Does not include sequences encoding the first portion of the signal peptide.

^10 ^nd, no significant similarity found to any *Triticum aestivum *sequences.

^11 ^na, not applicable.

**Figure 1:**
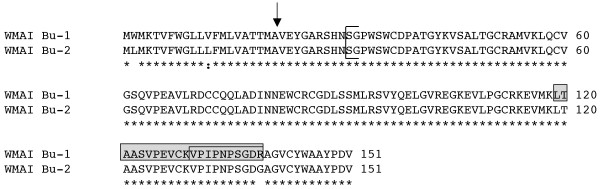
**Monomeric alpha-amylase inhibitors expressed in Butte 86**. In Clustal W2 sequence alignments in Figs. 1, 2, 3, 4 and 5, identical residues are denoted with asterisks, conserved substitutions with colons and semi-conserved substitutions with periods and predicted signal peptide cleavage sites are indicated by the arrow. The N-terminus of a WMAI determined by Kashlan and Richardson [21] is shown by a bracket. Unique peptides that distinguish WMAI Bu-1 from WMAI Bu-2 are enclosed in shaded boxes. Total MS/MS coverages of WMAI Bu-1 in protein spots 289 and 528 from Dupont et al. [17] were 61 and 64%, respectively (Additional file 5).

**Figure 2:**
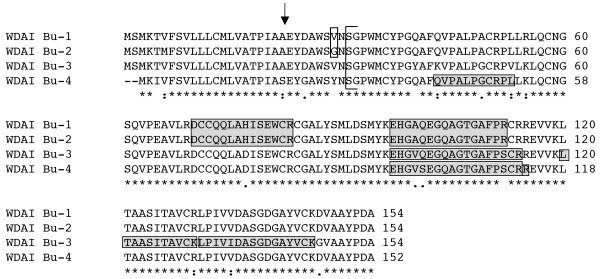
**Dimeric alpha-amylase inhibitors expressed in Butte 86**. N-termini of 0.53 and 0.19 type WDAI determined by Maeda et al. [24,25] are indicated by the bracket. The single amino acid difference between WDAI Bu-1 and WDAI Bu-2 is enclosed in a box. Peptides obtained by MS/MS that distinguish individual WDAI are enclosed in shaded boxes. Total MS/MS coverage of WDAI Bu-1 and WDAI Bu-2 in protein spot 312 from Dupont et al. [17] was 74%, coverage of WDAI Bu-3 in protein spot 283 was 68%, and coverage of WDAI Bu-4 in spots 286 and 283 was 77% and 60%, respectively (Additional file 5).

**Figure 3:**
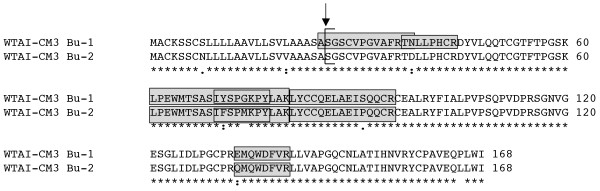
**CM3 subunits of the tetrameric alpha-amylase inhibitors expressed in Butte 86**. The N-terminus of a WTAI-CM3 inhibitor determined by Shewry et al. [27] is denoted by the bracket. Peptides obtained by MS/MS that distinguish individual WTAI-CM3 proteins are enclosed in shaded boxes. Total MS/MS coverages of WTAI-CM3 Bu-1 and WTAI-CM3 Bu-2 in protein spot 264 from Dupont et al. [17] were 90 and 76%, respectively (Additional file 5).

**Figure 4:**
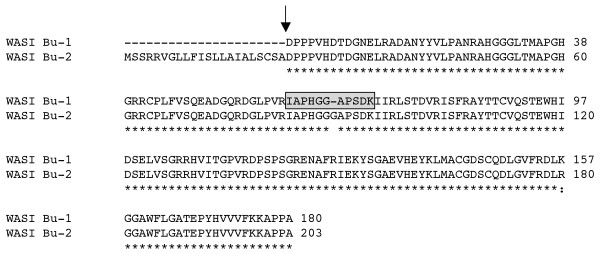
**Endogenous alpha-amylase inhibitors (WASI) expressed in Butte 86**. The peptide obtained by MS/MS that distinguishes WASI Bu-1 from WASI Bu-2 is enclosed in a shaded box. Total MS/MS coverage of WASI Bu-1 in protein spot 244 from Dupont et al. [17] was 73% (Additional file 5).

**Figure 5:**
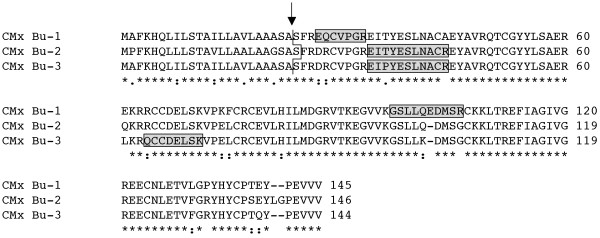
**CMx trypsin inhibitors expressed in Butte 86**. Differential signal peptide cleavage sites for the CMx inhibitors are indicated with the arrow and zig-zag line. Peptides obtained by MS/MS that distinguish individual CMx proteins are enclosed in shaded boxes. Total MS/MS coverage of CMx Bu-1 in protein spot 280 from Dupont et al. [17] was 36%, CMx Bu-2 in spot 290 was 57% and CMx Bu-3 in spot 281 was 60% (Additional file 5).

The sequences of 329 tryptic, chymotryptic and thermolytic peptides that identified protein spots from Butte 86 flour as amylase/protease inhibitors in the 2-DE analysis of Dupont et al. [17] were extracted from the original dataset. Among these were 134 distinct peptides, 20 obtained with chymotrypsin, 23 with thermolysin and 91 with trypsin (Additional file 4). All but four of the peptides were assigned to protein sequences deduced from Butte 86 contig consensus sequences. Between two and 17 peptides were assigned to each Butte 86 protein sequence (Table 2). Peptides that were unique for 16 of the encoded proteins were identified and made it possible to discriminate the products of closely related genes (Table 2, Figures 1, 2, 3, 4, 5, Additional file 4). Table 2 also compares identifications of protein spots reported in Dupont et al. [17] to assignments made to Butte 86 sequences.

**Table 2:** Association of Butte 86 contigs with alpha-amylase/protease inhibitors identified by MS/MS in a previous proteomic analysis of Butte 86 flour

Butte 86 Contig	# Peptides^1^	# Unique Peptides^2^	Spot Number^3^	MS/MS ID^4^
WMAI Bu-1	11	2	289*, 528*	[PRF:223520]^5^
WMAI Bu-2	9	0	nd	na
WDAI Bu-1/WDAI Bu-2^6^	9	2	280, 283, 285, 312*, 313	[SwissProt:P01085]^5^
WDAI Bu-3	9	3	281, 283*	TC11_338524
WDAI Bu-4	12	3	283, 286*	[Genbank:AAV91972]
WTAI-CM1 Bu-1	6	3	312, 313*	TC11_340510
WTAI-CM2 Bu-1	9	5	280*, 285*	[SwissProt:P16851]
WTAI-CM3 Bu-1	16	6	264*, 265*, 272	[SwissProt:P17314]
WTAI-CM3 Bu-2	14	4	264, 265	RS_UWI_15430
WTAI-CM16 Bu-1	17	12	266, 282, 284*, 286*	[SwissProt:P16159]
WTAI-CM17 Bu-1	11	6	274*, 282*	[Genbank:CAA42453]
WASI Bu-1^7^	12	1	244*	[SwissProt:P16347]^5^
WASI Bu-2	11	0	nd	na
CMx Bu-1	8	2	280, 285, 313	TC11_320696
CMx Bu-2	12	1	281, 286, 290*	TC11_308146
CMx Bu-3	14	2	281*, 290	TC11_309398
WTI Bu-1^7^	2	2	286^8^, 278	TC11_315743
WCI Bu-1	6	6	277*, 278*	[Genbank:CAD19440]

^1 ^Number of distinct peptides from Dupont et al. [17] that could be assigned to the protein encoded by Butte 86 contig.

^2 ^Number of peptides from Dupont et al. [17] that could be assigned exclusively to the protein encoded by Butte 86 contig.

^3 ^Spot numbers from 2-DE analysis of Dupont et al. [17]. * indicates that the sequence was the predominant protein identified in the spot. nd indicates that unique peptides for the protein encoded by the contig were not detected in any spots.

^4 ^MS/MS ID reported in Dupont et al. [17]. Accession numbers from DFCI Wheat Gene Index Release 11.0 are denoted with the prefix TC11_ and from HarvEST 1.14 are denoted with RS_UWI_. na indicates not applicable.

^5 ^Protein sequence determined by Edman degradation.

^6 ^Mature proteins encoded by WDAI Bu-1 and WDAI Bu-2 are identical.

^7 ^Missing all or part of coding region for signal sequence.

^8 ^Spot may contain an additional form of WTI not represented in Butte 86 EST collection.

### Monomeric alpha-amylase inhibitors

Fourteen Butte 86 ESTs assembled into two genes encoding monomeric alpha-amylase inhibitors (Table 1, Additional files 1, 2). Eleven ESTs comprised WMAI Bu-1, while three ESTs comprised WMAI Bu-2, suggesting that there is differential expression of the two genes in the endosperm. There are 16 bp differences in the coding regions of WMAI Bu-1 and WMAI Bu-2. These differences result in only three amino acid changes in the proteins, two in the signal peptide and a substitution of arginine for glycine near the carboxyl end of the mature protein (Figure 1). This substitution is one of five amino acid changes observed by Wang et al. [15] among proteins encoded by 73 WMAI genes from *T. aestivum *cv. Chinese Spring aneuploid lines and diploid progenitors of common wheat. The signal peptide cleavage site predicted by the Signal P algorithm [20] in the Butte 86 proteins is after the alanine at position 21. However, N-terminal sequencing of a WMAI protein from the variety Flanders [21] suggests that the mature protein begins with the serine residue at position 31 (Figure 1). This implies that the N-terminal region of the protein undergoes additional processing, most likely by an asparaginyl endopeptidase. Similar processing at the N-terminus has been observed for some omega gliadins [22] and has been proposed for certain low-molecular-weight glutenin subunits [23]. The predicted pIs of the two Butte 86 monomeric inhibitors differ substantially, 6.19 for the protein encoded by WMAI Bu-1 and 5.37 for the protein encoded by WMAI Bu-2.

Eleven peptides in the dataset of Dupont et al. [17] could be assigned to the Butte 86 WMAI proteins. Of these, nine were common to both WMAI Bu-1 and WMAI Bu-2 and two were found only in WMAI Bu-1 (Table 2, Additional file 4). In the Dupont et al. [17] study, two protein spots of similar size but different pI (289, 528) were identified by MS/MS as WMAI [PRF:223520] (Table 2). While it might be expected that the more acidic protein (528) would correspond to WMAI Bu-2, peptides unique to WMAI-2 were not found. Instead, two overlapping tryptic peptides unique to WMAI-Bu-1, LTAASVPEVCKVPIPNPSGDR and VPIPNPSGDR, were identified for both spots (Figure 1).

### Dimeric alpha-amylase inhibitors

The dimeric alpha-amylase inhibitors are represented by 10 Butte 86 ESTs (Table 1, Additional files 1, 2). WDAI-Bu-1, composed of five ESTs, and WDAI-Bu-2, composed of two ESTs, encode 0.19 type WDAI proteins. At the DNA level, there are five bp differences between the two coding regions. These result in only two amino acid differences in the proteins, one in the signal peptide and the other at position 29 (Figure 2). While the signal peptide cleavage site predicted by Signal P is after the alanine at position 22 in the Butte 86 proteins, N-terminal amino acid sequencing of a similar 0.19 type WDAI protein by Edman degradation [24] suggests that the mature protein begins with the serine residue at position 31 (Figure 2), implying that these WDAI proteins, like the WMAI proteins, undergo further processing. Thus, the mature proteins encoded by the two gene sequences are identical.

WDAI Bu-3, composed of two ESTs, and WDAI Bu-4, represented by only one EST, encode the 0.53 type WDAI proteins (Table 1, Additional files 1, 2). WDAI Bu-4 is missing 6 bp at the 5' end of the coding region. There are 23 bp differences in the remainder of the coding regions of the two sequences, resulting in two amino acid changes in the signal peptides and nine amino acid changes in the mature proteins (Figure 2). While the proteins have similar MWs, they differ in pI from each other and from the 0.19 type WDAI (Table 1). Similar to the 0.19 type dimeric inhibitors, the signal peptide cleavage site predicted by Signal P is after the alanine at position 22 in the Butte 86 proteins, but N-terminal sequencing of a similar 0.53 type WDAI protein by Edman degradation [25] suggests that the mature proteins begin with the serine residue at position 31 (Figure 2).

Of the 17 peptides in the dataset that could be assigned to Butte 86 WDAI, two peptides, DCCQQLAHISEWCR and EHGAQEGQAGTGAFPR, were unique to the identical proteins encoded by WDAI Bu-1 and WDAI Bu-2. Three peptides, EHGVQEGQAGTGAFPSCR, LTAASITAVCK and LPIVIDASGDGAYVCK were unique to the protein encoded by WDAI Bu-3, and three peptides, QVPALPGCRPL, EHGVSEGQAGTGAFPSCR and EHGVSEGQAGTGAFPSCRR, were unique to the protein encoded by WDAI Bu-4 (Table 2, Figure 2, Additional file 4). The proteomics data clearly support the existence of WDAI Bu-4, although a single EST represents this dimeric inhibitor. In the study of Dupont et al. [17], seven protein spots (280, 281, 283, 285, 286, 312, 313) that differed in charge but not MW contained WDAI identified as one of three distinct sequences, [SwissProt:P01085], TC11_338524 or [GenBank:AAV91972] (Table 2).

### Tetrameric alpha-amylase inhibitors

Fifty-eight ESTs representing six different coding regions correspond to tetrameric alpha-amylase inhibitors (Table 1, Additional files 1, 2). By far the most abundant sequence was WTAI-CM3 Bu-1, represented by 20 ESTs. The protein encoding WTAI-CM3 Bu-1 was identical to CM3 proteins characterized previously in both bread and durum wheat [26]. A similar sequence, WTAI-CM3 Bu-2, is represented by seven ESTs and has not been reported before. There are 24 bp differences in the coding regions of WTAI-CM3 Bu-1 and WTAI-CM3 Bu-2. These result in two amino acid changes in the signal peptide and six amino acid changes in the mature protein (Figure 3). WTAI-CM3 Bu-1 and WTAI-CM3 Bu-2 have identical pIs and very similar predicted MWs that are greater than other subunits of the WTAI.

Of the 20 peptides in the dataset that could be assigned to Butte 86 WTAI-CM3 type sequences, six were unique to WTAI-CM3 Bu-1 and four were unique to WTAI-CM3 Bu-2 (Table 2, Figure 3, Additional file 4). Three 2-DE spots (264, 265, 272) of similar MW but different pI were identified as the CM3 type sequences [SwissProt:P17314] and RS_UWI_15430 in the study of Dupont et al. [17]. Signal P predicts that the signal peptide cleavage site of WTAI-CM3 Bu-1 would be after the alanine at position 25, consistent with the N-terminal protein sequence of a CM3-type protein reported by Shewry et al. [27]. However, the MS/MS identification of the peptide ASGSCVPGVAFRTN for WTAI-CM3 Bu-1 suggests an alternate signal peptide cleavage site in this protein (Figure 3).

WTAI-CM16 Bu-1 is another abundant sequence in Butte 86 developing grain, represented by 10 ESTs. The Butte 86 sequence is identical to cDNAs characterized previously in both bread wheat and durum wheat [28,29]. WTAI-CM17 Bu-1 is a closely related protein that is represented by five Butte 86 ESTs. The protein encoded by the Butte 86 sequence differs from the CM17 protein reported previously [30] by a single amino acid change in the signal peptide. The proteins encoded by WTAI-CM16 Bu-1 and WTAI-CM17 Bu-1 are more acidic than the other WTAI subunits, with pIs of 5.02 and 4.87, respectively. Seventeen peptides in the dataset could be assigned to WTAI-CM16 Bu-1 and 12 were found only in this protein. Eleven peptides could be assigned to WTAI-CM17 Bu-1 and six were exclusive to this protein. In the study of Dupont et al. [17], WTAI-CM16 [SwissProt:P16159] was the predominant protein identified in two protein spots with similar pIs but different MWs (284, 286) and a minor component of two other spots (266, 282). WTAI-CM17 [GenBank:CAA42453] was the predominant protein in two spots (274, 282) (Table 2).

Butte 86 also expresses genes encoding CM1 and CM2 proteins. Each of these genes is represented by eight ESTs (Table 1). WTAI-CM1 Bu-1 encodes a protein that contains a single amino acid difference from one reported previously in bread wheat [26], a proline in place of a serine 20 amino acids from the C-terminus (Additional file 3). WTAI-CM2 Bu-1 encodes a protein that is identical to ones reported previously in bread and durum wheat [31] (Additional file 3). Six peptides in the dataset could be assigned to WTAI-CM1 Bu-1 and nine peptides could be assigned to WTAI-CM1 Bu-2. It is notable that a chymotryptic peptide VTPGHcNVm contained the proline substitution that was found in WTAI-CM1 Bu-1. This peptide is also found in WTAI-CM2 Bu-1. A tblastn search of the NCBI EST database with VTSGHCNVM, the peptide found in the Garcia-Maroto et al. CM1 sequence [26], failed to find any translated ESTs with a serine in the third position of this peptide (tblastn parameters: non-human, non-mouse ESTs limited to *Triticum*, expect 30,000, BLOSUM62, no compositional adjustments, no filters or masks, database last searched on 5/16/11). In the Dupont et al. [17] study, two protein spots (312, 313) of similar molecular weight but different pI were identified as WTAI-CM1 encoded by the contig TC11_340510 and another two spots (280, 285) were identified as WTAI-CM2 [SwissProt:P16851] (Table 2).

### Endogenous alpha-amylase inhibitors

EST analysis revealed that two distinct sequences for endogenous alpha-amylase inhibitors are expressed in Butte 86 grain. WASI Bu-1 was represented by two ESTs while WASI Bu-2 was represented by three ESTs (Table 1, Additional files 1, 2). The coding regions of WASI Bu-1 and WASI Bu-2 differ by 15 bp and one 3 bp indel. Surprisingly, these changes result in only one conservative substitution, an arginine for a lysine 24 amino acids from the C-terminus of the protein and an extra glycine in the center of WASI Bu-2 (Figure 4). The protein encoded by WASI Bu-1 is identical to one characterized by Maeda [32] from bread wheat flour. Only one cDNA for WASI has been reported previously from cv. Chinese Spring and this cDNA is similar, but not identical to WASI Bu-2. Eleven peptides in the dataset could be assigned to both WASI Bu-1 and WASI Bu-2 (Table 2, Additional file 4). An additional tryptic peptide, IAPHGGAPSDK, is exclusive to WASI Bu-1. In the study by Dupont et al. [17], only one protein spot (244) was identified as the endogenous inhibitor [SwissProt:P16347]. The data indicates that the spot corresponds to the WASI Bu-1 sequence, but does not rule out the presence of WASI Bu-2 that has a similar size and pI.

### Protease inhibitors

Seven ESTs corresponding to CMx trypsin inhibitors were identified in Butte 86 (Table 1, Additional files 1, 2). These represent three different expressed genes, CMx Bu-1, CMx Bu-2 and CMx Bu-3, none of which have been reported previously. At the DNA level, CMx Bu-2 is most similar to a cDNA sequence identified by Sanchez de la Hoz et al. [4] that contains a premature stop codon (Table 1). There are 30 bp differences and two indels between CMx Bu-1 and CMx Bu-2 and 23 bp differences and one indel between CMx Bu-2 and CMx Bu-3. The protein encoded by CMx Bu-2 contains five amino acids in the signal peptide and nine amino acids in the mature protein that differ from the protein encoded by the cDNA from Sanchez de la Hoz et al., allowing for read-through of the stop codon [4]. In addition to the changes in the signal peptide, there are eight amino acid changes and two indels in the CMx Bu-1 protein and seven amino acid changes and one indel in the CMx Bu-3 protein relative to the CMx Bu-2 protein (Figure 5). Signal P predicts a slightly different signal peptide cleavage site in CMx Bu-2 than in the other two Butte 86 proteins. The predicted molecular weights of the mature proteins are similar to the WMAI, WDAI, and WTAI subunits CM1, CM2, CM16 and CM17 from Butte 86. However, the pIs of proteins encoded by CMx Bu-2 and CMx Bu-3 are more basic than the other inhibitors.

Eight peptides in the dataset could be assigned to CMx Bu-1, 12 peptides to CMx Bu-2, and 14 peptides to CMx Bu-3 (Table 2, Additional file 4). Two peptides were found exclusively in CMx Bu-1 and one peptide was only found in CMx Bu-2. Although only one EST was found for CMx Bu-3, the existence of this protein was supported by two unique peptides (Figure 5). Three CMx proteins were distinguished by MS/MS in six protein spots (280, 281, 285, 286, 290, 313) from Butte 86 flour in the study of Dupont et al. [17] and identified as TC11_320696, TC11_308146 and TC11_309398 (Table 2). None of these proteins have been characterized previously in wheat.

A single EST, BQ806495, represents another putative trypsin inhibitor from Butte 86 but is missing a portion of the 5' end encoding the signal peptide. A blastn search of NCBI nr database found no similar sequences to this EST. However, the protein encoded by the EST has weak similarity to trypsin inhibitor CMe from barley [GenBank:X17302] and is referred to here as WTI Bu-1. Like many of the other inhibitors, the protein encoded by WTI Bu-1 has 10 cysteines in an arrangement similar to the other alpha-amylase/protease inhibitors. Two peptides in the dataset could be assigned to WTI Bu-1. Two other peptides found in the dataset, DTQQTAPTPGK and AYVVQQTcK, differ from WTI Bu-1 by a two amino acid indel and a substitution, respectively, suggesting that there may be an additional form of WTI in Butte 86 that was not represented in the EST collection. In the study of Dupont et al. [17], two protein spots (278, 286) contained proteins identified as TC11_315743, but TC11_315743 was not the predominant protein in either spot (Table 2).

Two ESTs were identified that are similar to a cDNA [GenBank:AJ422078] from the hexaploid wheat cv. San Pastore that encodes WCI, a protein that reportedly inhibits mammalian, insect and endogenous chymotrypsin (Table 1, Additional files 1, 2). The protein encoded by WCI Bu-1 differs from that encoded by [GenBank:AJ422078] in that it contains a methionine in place of an isoleucine three amino acids from the predicted N-terminus of the mature protein. Six peptides in the dataset could be assigned to WCI Bu-1 (Table 2). Two spots (277, 278) were identified as WCI [GenBank:CAD19440] in Butte 86 flour in the study of Dupont et al. [17] (Table 2).

### Comparison of numbers of ESTs to protein accumulation levels

**Figure 6:**
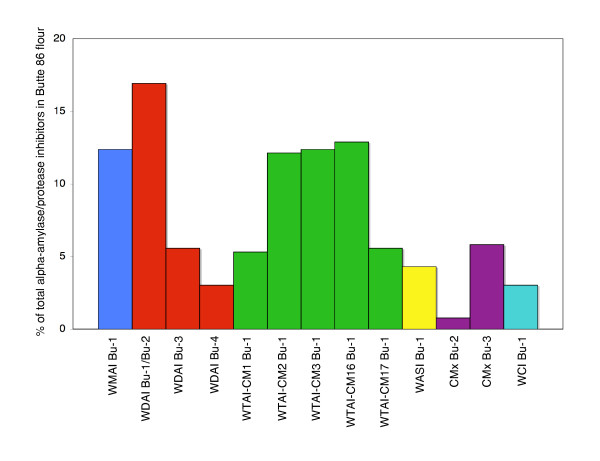
**Accumulation of alpha-amylase/protease inhibitors in Butte 86 flour**. The amount of individual inhibitors as a percentage of total alpha-amylase/protease inhibitor protein in Butte 86 is summarized from proteomic analysis of Dupont et al. [17]. Protein spots indicated with * in Table 2 for each inhibitor type were included in the analysis.

Since the cDNA library used to generate the ESTs was made from a mix of RNA from multiple developmental time points from grain grown under six different regimens of temperature, fertilizer and water [33], the number of ESTs should reflect the abundance of transcripts corresponding to each gene during grain development. In this study, 97 ESTs were assembled into 19 alpha-amylase/protease inhibitor sequences and the most abundant sequence was represented by 20 ESTs. In a survey of alpha gliadin ESTs from the same cDNA library, a similar number of ESTs were divided among 19 genes and the most abundant alpha gliadin was represented by 15 ESTs [34]. Thus, the EST data suggest that transcripts for genes within the alpha-amylase/protease inhibitor family are present in developing grain at levels that are similar to some of the major gluten proteins. The EST data also indicate that transcripts for genes within the WTAI group are quite abundant and that there are differences in the abundance of transcripts among individual members. Transcripts for WMAI Bu-1 were also abundant. Transcripts for WDAI Bu-1/Bu-2 encoding the 0.19 type dimeric inhibitors were more prevalent than those for WDAI Bu-3 and WDAI Bu-4 encoding the 0.53 type inhibitors. Figure 6 shows the relative proportions of amylase/protease inhibitors in Butte 86 flour determined in the proteomic analysis of Dupont et al [17]. The most abundant proteins were WDAI Bu-1/Bu-2, represented by eight ESTs and WMAI Bu-1, represented by 11 ESTs. Among the tetrameric inhibitors, WTAI-CM2 Bu-1, WTAI-CM3 Bu-1 and WTAI-CM16 Bu-1 were accumulated to similar levels and were two-fold more abundant than WTAI-CM1 Bu-1 and WTAI-CM17 Bu-1, although there were more than two times the number of ESTs for WTAI-CM3 than for the other WTAI subunits. CMx Bu-3 was represented by a single EST, but this protein was more abundant than CMx Bu-2 that was represented by four ESTs.

## Conclusions

This is the first study to identify the majority of low molecular weight alpha-amylase/protease inhibitors in a single cultivar of bread wheat. Coding sequences for 19 alpha-amylase/protease inhibitors expressed in grain from the US wheat Butte 86 were identified by EST analysis. The study revealed DNA sequences for several inhibitors not described previously, identified genes with substantial sequence differences that encode very similar proteins, and made it possible to associate specific gene sequences with individual proteins in a comprehensive proteomic map from the same cultivar. Knowledge about the sequences of genes encoding alpha-amylase/protease inhibitors will facilitate further studies on the expression of individual genes in grain produced under different conditions and make it possible to manipulate the composition of alpha-amylase/protease inhibitors in plants using either breeding or biotechnology approaches. Such studies will better define the roles of individual proteins in plant defense mechanisms, abiotic stress responses and human allergies.

## Methods

ESTs for alpha-amylase/protease inhibitors from *Triticum aestivum *'Butte 86' were downloaded from NCBI (Additional file 1). All ESTs were from a cDNA library produced from equal amounts of RNA prepared at two to four day intervals between anthesis and maturity from Butte 86 developing grain grown under six separate environmental regimens [33]. Environmental regimens included 24°C days/17°C nights with and without post-anthesis fertilizer, 37°C days/17°C nights with and without post-anthesis fertilizer, and 37°C days/17°C nights plus drought with and without post-anthesis fertilizer. ESTs were assembled with Lasergene Seqman Pro (DNASTAR, Inc., Madison, WI) using the Classic Assembler with default settings except that the minimum match percentage was set to 98 and the minimum sequence length was set to 50. Assemblies were inspected manually and mismatches that occurred in overlap regions of ESTs were resolved by examining phred quality scores for individual ESTs as detailed in Altenbach et al. [35]. The suffix "Bu-x", where × represents the number of a sequence within a particular group, was added to the name of the inhibitor to designate that the sequence was derived from Butte 86. DNA consensus sequences (shown in Additional file 2) were translated using functions within the Lasergene software. Cleavage of signal peptides was predicted using SignalP 3.0 Server [20]. MWs and pIs of deduced proteins were calculated using ProtParam found on the ExPASy Proteomics Server [36]. Sequence alignments were performed using ClustalW2 with default settings [37]. DNA and protein homology searches were performed using blastn, tblastn and blastp from NCBI [38].

Proteins separated by 2-DE from Butte 86 flour were identified by MS/MS in Dupont et al. [17]. Peptides from all protein spots identified as low molecular weight alpha-amylase/protease inhibitors in the Dupont et al. [17] study were extracted from Scaffold Version 3.00.02. Unique peptides within the dataset were identified and used to search against the sequences of alpha-amylase/protease inhibitors deduced from Butte 86 contig consensus sequences (Additional file 4). Peptides not found within Butte 86 protein sequences were used to search the NCBI EST database (non-human, non-mouse EST, limited to *Triticum*) to determine whether there were other ESTs that could be associated with the peptide. Association of original MS/MS data from Dupont et al. [17] with Butte 86 alpha-amylase/protease inhibitor sequences reported in this manuscript is detailed in Additional file 5. The entire MS/MS dataset from Dupont et al. [17] can be downloaded from ProteomeCommons.org Tranche using the hash: hCc5INiKGH0m4DEfxLbShm1F+us+JyZ/HENjkOTlGcni8NmnyoEwU5i7Onf/Po2kNtnP10SCdgODD6Swo0hgF69d3dIAAAAAAAB6hg==

## Abbreviations

2-DE: 2-dimensional gel electrophoresis; EST: expressed sequence tag; MS/MS: tandem mass spectrometry; MW: molecular weight; NCBI: National Center for Biotechnology Information; WDAI: wheat dimeric amylase inhibitor; WMAI: wheat monomeric amylase inhibitor; WTAI: wheat tetrameric amylase inhibitor, WASI: wheat amylase subtilisin inhibitor.

## Competing interests

The authors declare that they have no competing interests.

## Authors' contributions

SA assembled contigs, analyzed gene sequences and drafted the manuscript. WV and FD contributed to proteomic data. All authors have read and approved the manuscript.

## Supplementary Material

Additional file 1 **Assignment of ESTs from Butte 86 to contigs encoding alpha-amylase/protease inhibitors**. Click here for file

Additional file 2 **DNA consensus sequences of Butte 86 contigs for alpha-amylase/protease inhibitors**. Click here for file

Additional file 3 **Amino acid sequences of alpha-amylase/protease inhibitors deduced from consensus sequences of Butte 86 contigs**. Click here for file

Additional file 4 **Unique tryptic, chymotryptic and thermolytic peptides used to identify alpha-amylase/protease inhibitors in 2-DE/MS/MS study of Butte 86 flour protein by Dupont et al**. [17]**and assignment of peptides to protein sequences deduced from Butte 86 contig consensus sequences**. Click here for file

Additional file 5 **Association of MS/MS data from Dupont et al.**[17]**with Butte 86 alpha-amylase/protease inhibitor sequences**. Click here for file
