# Supplementary material for: The spectrum of low molecular weight alpha-amylase/protease inhibitor genes expressed in the US bread wheat cultivar Butte 86
Source: BMC Res Notes. 2011 Jul 20;4:242. doi: 10.1186/1756-0500-4-242 (PMC3154163; doi:10.1186/1756-0500-4-242)
Supplement: Additional file 2 — DNA consensus sequences of Butte 86 contigs for alpha-amylase/protease inhibitors. [file 1756-0500-4-242-S2.DOC]

Additional file 2. DNA consensus sequences of Butte 86 contigs for alpha-amylase/protease inhibitors. Coding regions are underlined.

WMAI Bu-1

TTCGGCACGAGGTAAGATAGTACTAAATTGAAAAATGTGGATGAAGACCGTGTTCTGGGGGCTCCTAGTATTCATGCTCGTGGCGACAACAATGGCGGTCGAGTATGGTGCAAGGAGCCATAACAGTGGTCCTTGGAGTTGGTGCGATCCGGCGACGGGCTACAAGGTGAGCGCACTCACGGGCTGCCGGGCAATGGTGAAGCTCCAGTGTGTGGGCAGTCAGGTGCCCGAGGCTGTCCTAAGAGATTGCTGCCAGCAGCTGGCCGACATCAACAACGAATGGTGCAGGTGCGGGGACCTCAGCAGCATGTTGCGTAGTGTTTATCAGGAGCTCGGCGTGCGTGAGGGGAAGGAGGTGCTCCCAGGTTGCCGGAAGGAGGTGATGAAGCTCACGGCGGCGAGCGTGCCTGAGGTCTGCAAGGTGCCCATTCCCAACCCGTCGGGAGACAGAGCAGGTGTCTGCTACTGGGCCGCGTATCCGGACGTCTAGTCAAGCGAATCTGCATCTTAATTGGTGCGGTGCGTGCGCACAAGATAAATAAAATTANAGTAATGGTGCGGTGCGTGCGCACAAGATAAATAAAATTAAGTAATGGTGTATGTGCCTCTGCGAGATGCATGGGTCATGAATAAATGTGAACTTTGTTTCCTGTTGATGGAT

WMAI Bu-2

TCGGCACGAGGCTAAATTGAAACATGTTGATGAAGACCGTGTTCTGGGGGCTCCTACTATTCATGCTCGTGGCGACAACAATGGCGGTCGAGTACGGTGCAAGGAGTCATAACAGTGGTCCTTGGAGTTGGTGCGATCCGGCCACGGGCTACAAGGTGAGCGCGCTCACGGGCTGCCGGGCAATGGTGAAGCTCCAGTGTGTGGGCAGTCAGGTGCCCGAGGCTGTCCTAAGAGATTGCTGCCAGCAGCTCGCCGACATCAACAACGAGTGGTGCAGGTGCGGGGACCTCAGCAGCATGTTGCGCAGTGTGTATCAGGAACTCGGCGTGCGTGAGGGAAAGGAGGTGCTCCCAGGTTGTCGAAAGGAGGTGATGAAGCTCACGGCGGCGAGCGTGCCTGAGGTCTGCAAGGTGCCCATTCCCAACCCGTCGGGAGACGGAGCAGGTGTCTGCTACTGGGCGGCGTATCCGGACGTCTAGTCAAGCGAATCTGCATCTTAATTGGTGCGGTGCGTGCGCACAAGATAAATAAAATTAAGTAATGGTGTATGTGCCTCTGCGAGATGCATGGGTCATGAATAAATGTGAACTTAGTTTCCAAAAAAAAAAAAAAAAA

WDAI Bu-1

GAGGCAGAAAGTCTGTGCATAGAGGAAGAATGTCGATGAAGACCGTGTTCTCGGTGCTCCTACTATGTATGCTCGTGGCGACACCCATAGCAGCCGAGTACGACGCATGGAGCGTTAACAGTGGTCCTTGGATGTGCTATCCGGGGCAGGCCTTCCAGGTTCCCGCGCTCCCTGCCTGTCGTCCATTGCTGAGGCTCCAGTGCAATGGCAGCCAGGTGCCCGAGGCTGTCCTAAGGGACTGCTGCCAGCAGCTCGCCCACATCAGCGAGTGGTGCAGGTGCGGGGCCCTCTACAGCATGTTGGACAGCATGTATAAGGAGCATGGCGCGCAGGAGGGACAGGCAGGGACAGGAGCGTTCCCACGCTGCCGGAGGGAGGTGGTGAAGCTGACGGCGGCGAGCATCACAGCGGTTTGCAGGCTACCCATCGTCGTTGATGCGTCCGGAGATGGAGCGTATGTCTGCAAGGATGTGGCCGCATACCCAGACGCCTAGTCAAGCAAATGAGTAGCTACTATGTTGTGTCATGCACCTACCTCTTAATTTGTGGGAGTGCGCACAAACTAAATAAAATGGTGTATGTGCCTATGCGAAATGCATTGGTAATGAATAAAGTGAAGTTCCCTGTTTGATCGAGAA

WDAI Bu-2

TACTAAATTAGAGGCAGAAAGTCTGTGCATAGAGGAAGAATGTCGATGAAGACCATGTTCTCGGTGCTCCTACTATGTATGCTCGTGGCGACACCCATAGCAGCCGAGTACGACGCATGGAGCGGTAACAGTGGTCCTTGGATGTGCTATCCGGGGCAGGCCTTCCAGGTGCCCGCGCTCCCCGCCTGTCGTCCATTGCTGAGGCTCCAGTGCAATGGCAGCCAGGTGCCCGAGGCTGTCCTAAGGGACTGCTGCCAGCAGCTCGCCCACATCAGCGAGTGGTGCAGGTGCGGGGCCCTCTACAGCATGTTGGACAGCATGTATAAGGAGCATGGCGCGCAGGAGGGACAGGCAGGGACAGGAGCGTTCCCACGCTGCCGGAGGGAGGTGGTGAAGCTGACGGCGGCGAGCATCACAGCGGTCTGCAGGCTACCCATCGTCGTTGATGCGTCCGGAGATGGAGCGTATGTCTGCAAGGATGTGGCCGCATACCCAGACGCCTAGTCAAGCAAATGAGTAGCTACTATGTTGTGTCATGCACCTGCCTCTTAATTTGTGGGAGTGCGCACAAACTAAATAAAATGGTGTATGTGCCTATGCGAAATGCATTGTAATGAATAAAAGTGAAGTTCCCTG

WDAI Bu-3

TTCGGCACGAGGGTTAATATAGTACTAAATCAGAGATAGAAAGTCTGTGCGCAGAGGAACAATGTCGATGAAGACCGTGTTCTCGGTGCTCCTGCTATGTATGCTCGTGGCGACACCCATAGCGGCCGAGTACGACGCATGGAGCGTTAACAGTGGTCCCTGGATGTGCTATCCAGGGTATGCCTTTAAGGTGCCAGCGCTCCCTGGCTGTCGTCCAGTGCTGAAGCTCCAGTGCAATGGCAGCCAGGTGCCCGAGGCTGTCCTAAGGGACTGCTGCCAGCAGCTCGCCGACATCAGCGAGTGGTGCAGGTGCGGTGCCCTCTACAGCATGTTGGACAGCATGTATAAGGAGCATGGCGTGCAGGAGGGACAGGCGGGGACAGGCGCGTTCCCAAGCTGCCGGAGGGAGGTGGTGAAGCTGACGGCGGCGAGCATCACGGCGGTCTGCAAGCTACCCATCGTCATTGATGCGTCTGGAGATGGAGCGTATGTCTGCAAGGGTGTGGCCGCATACCCGGACGCCTAGTCAAGCGAATGAGTAGCTACTATGTTGTGTCATGCATCTGCCTCTTAATTTGTGGGGTGCGCACAAGTTGAATAAAATGGTGTATGCGCCTATGCGAAATGCATCATTAATGAATAAAAGCGAAGTTCCCTGTTGATCGAGAAAAAAAAAA

WDAI Bu-4

TTCGGCACGAGGCTAAATCAGAGACAGAAAGTCTGTTCGCAGAGGAACAAAGTCGATGAAGATCGTGTTCTCGGTGCTCCTGCTATGTATGCTCGTGGCGACACCCATAGCGTCCGAGTACGGCGCATGGAGCTATAACAGTGGTCCCTGGATGTGCTATCCGGGGCAGGCCTTCCAGGTGCCCGCGCTCCCTGGCTGTCGTCCATTGCTGAAGCTCCAGTGCAATGGCAGCCAGGTGCCCGAGGCTGTCCTAAGGGACTGCTGCCAGCAGCTCGCCGATATCAGTGAGTGGTGCAGGTGCGGTGCCCTCTACAGCATGTTGGATAGCATGTATAAGGAGCATGGCGTGTCGGAGGGACAGGCGGGGACAGGCGCATTCCCAAGCTGCCGGAGGGAGGTGGTGAAGCTGACGGCGGCGAGCATCACGGCGGTCTGCAGGCTACCCATCGTCGTTGATGCGTCCGGAGATGGAGCGTATGTCTGCAAGGATGTGGCCGCATACCCGGATGCCTAGTCAAGCGAATGAGTAGCTAATATGTTGTGTGATACATCTGCCTCTTAATTTATGGGGTGCGCACAAGCTGAATAAAATGGTGTATGCACCTATGCGAAATGCATCGTTAATGAATAAAAACGAAGTTCCCTGTTGATAAAAAAAAAAAAAAAAAA

WTAI-CM1 Bu-1

TCGGCACGAGGGTGCAGTGCACAACTCAAGAAACTACACCAACAAACCGGACAAGGCTAGAAAGAATAACATGGCGTCCAAGTCTAGCATCTCCCCCCTCCTCTTGGCCACCGTCCTGGTCTCCGTCTTCGCCGCCGCCACAGCCACAGGTCCATATTGCTACGCCGGGATGGGCCTTCCGATCAACCCGCTTGAAGGCTGCCGGGAGTATGTCGCACAGCAAACCTGTGGCATCAGCATATCCGGGTCGGCGGTGTCCACCGAGCCGGGGAACACCCCAAGGGATCGGTGCTGCAAGGAGCTTTACGACGCCTCGCAGCATTGCCGGTGCGAGGCAGTGCGCTACTTCATAGGGCGGAGGTCTGATCCCAATTCCAGCGTGCTCAAGGACCTCCCCGGATGCCCCAGGGAGCCCCAGAGGGACTTCGCCAAGGTGCTCGTTACGCCGGGGCACTGCAACGTGATGACCGTTCACAACGCCCCATACTGCCTCGGTTTGGACATATAAAGATAGATAGATCCGTCGCCCATGAATGAATAAGCATGCTCCGTCCGTGGATGTGTGGCATGCATATATGCATATGTGAGCTCCCGTTGCTCAACATTTGCTTTACTAATAAAGAGAATCATTTTGTGGTTCTTA

WTAI-CM2 Bu-1

ATTCGGCACGAGGCAGCACAGTGCACAACCTCAAGAAACTACACCAACAAACCGGACCAAGCTAGAAAGAATAATATGGCGTCCAAGTCTAGCATCACCCACCTCCTCTTGGCCGCCGTCCTGGTCTCCGTCTTCGCCGCCGCCGCAGCCACAGGCCCGTATTGCTACCCGGGGATGGGTCTTCCGAGCAACCCGCTTGAAGGCTGCCGGGAGTATGTCGCACAGCAAACTTGTGGCGTCGGCATCGTCGGGTCGCCGGTATCCACCGAGCCGGGGAACACCCCGAGGGATCGGTGCTGCAAGGAGCTTTACGACGCCTCGCAGCATTGCCGGTGCGAGGCGGTGCGCTACTTCATAGGGCGGACGTCTGATCCCAATTCCGGCGTGCTCAAGGACCTTCCCGGATGCCCCAGGGAGCCCCAGAGGGACTTCGCCAAGGTGCTCGTTACGCCGGGGCACTGCAACGTGATGACCGTTCACAACACCCCATACTGCCTCGGTTTGGACATATAAAGATGGATAGATCCGTCTCTCATGAATGAATAAGCATGTTCCATCCATGGATGTGTGGCATGCATATATATATGAACAAGAATAAAGGGAATCATTTTGTGATTCTTAAATTTCAACTCAATCTTTTGTTAAAAAAAAAAAAAAAAAA

WTAI-CM3 Bu-1

GAGGCACCAGCGAACCAGACTTGGCTAGAATACCATGGCGTGCAAGTCCAGCTGCAGCCTCCTCCTCTTGGCCGCCGTCCTGCTCTCCGTCTTGGCCGCTGCTTCCGCCTCCGGCAGCTGCGTCCCAGGGGTGGCTTTTCGGACCAATCTTCTGCCACACTGCCGCGACTATGTGTTACAACAAACTTGTGGCACCTTCACCCCTGGGTCAAAGTTACCCGAATGGATGACATCTGCGTCGATATACTCCCCTGGGAAACCGTACCTCGCCAAGTTGTATTGCTGCCAGGAGCTCGCAGAAATTTCTCAGCAGTGCCGGTGCGAGGCGCTGCGCTACTTCATAGCGTTGCCGGTACCGTCTCAGCCTGTGGACCCGAGGTCCGGCAATGTTGGTGAGAGCGGCCTCATCGATCTGCCCGGATGCCCCAGGGAGATGCAATGGGACTTCGTCAGATTACTCGTCGCCCCGGGGCAGTGCAACTTGGCGACCATTCACAATGTTCGATACTGCCCCGCCGTGGAACAGCCTCTGTGGATCTAGAGATAAAATCAGTCGCTCGTGAATAAGCATGCATGTTGCATCCATA

WTAI-CM3 Bu-2

ATTCGGCACGAGGGCGAACAGACTTGGCTAGAATACCATGGCGTGCAAGTCCAGCTGCAACCTCCTCCTCTTGGCCGCCGTCCTGCTCTCCGTCGTCGCCGCTGCTTCCGCCTCCGGCAGCTGCGTCCCAGGGGTGGCTTTTCGGACCGATCTTCTGCCACACTGCCGCGACTATGTGTTACAACAAACTTGTGGCACCTTCACCCCCGGGTCGAAGTTACCCGAATGGATGACATCCGCGTCAATATTCTCCCCCATGAAGCCATACCTCGCCAAGTTGTATTGCTGCCAGGAGCTCGCAGAAATTCCTCAGCAGTGCCGGTGCGAGGCGCTGCGCTACTTCATAGCGTTGCCGGTACCGTCTCAGCCCGTGGACCCGAGGTCCGGCAATGTCGGTGAGAGCGGCCTCATCGACCTGCCCGGATGCCCCAGGCAGATGCAATGGGACTTTGTCAGATTACTTGTTGCCCCGGGGCAGTGCAACTTGGCGACCATTCACAATGTTCGATACTGCCCCGCCGTGGAACAGCTTCTGTGGATCTAGAGATAAAATCAGTCGTTCGTGAATAAGCATGCATGTTGCATCCATAGGCGTGTGGTGTGCATGCATGCATATGTGAGCTCCGCGCGCTCAACATGTGTGGGCTATCTGCTATGAATGAGAATAAAGAGAACCATTTTGTGGTTCTTTATTT

WTAI-CM16 Bu-1

CCTATACCACGAACTGGGCCTGTCAAAAAAATATGGCGTCCAAGTCCAACTGCGTTCTCCTCTTGGCCGCCGTCCTAGTCTCCATCTTTGCCGCCGTTGCCGCCATCGGCAATGAAGATTGCACCCCATGGATGAGTACTCTGATCACTCCACTCCCAAGCTGCCGTGACTATGTGGAACAACAAGCATGTCGCATCGAAACGCCCGGGTCGCCGTACCTCGCCAAGCAGCAGTGCTGTGGGGAGCTTGCAAACATTCCGCAGCAGTGCCGATGCCAGGCGCTGCGCTACTTCATGGGGCCGAAGTCTCGTCCGGATCAGAGCGGCCTCATGGAACTCCCCGGATGCCCTAGGGAGGTGCAGATGGACTTCGTGAGGATACTCGTCACGCCGGGGTACTGCAACTTGACGACCGTTCACAACACTCCGTACTGCCTCGCTATGGAGGAGTCTCAGTGGAGCTAGAGACAATTCTCTCGCTCATGAATAAATAAGCATGTTGCGACCATACATGTGTGACATGCATATATGCATATAGGACGAGCTCCGCGCGCTCATCATGTGTGGTGCTATGTGCTACATATATGGATAAGAATAAAGGGAATCATTTTCCTTCTAAAAAAAAAAAAAAAAAAAAA

WTAI-CM17 Bu-1

ATTCGGCACGAGGGCAGTGCAAAAGAAAAAACCACACCAACGAACTTGGCCTCCATCCAAAAATATGGCGTCCAAGTCCAACTACAATCTCCTCTTCGCGGCCCTCCTAGTCTTCATCTTTGCCGCCGTTGCCGCCGTCGGCAATGAAGATTGCACCCCATGGACGAGTACTCTGATCACTCCACTCCCAAGCTGCCGTAACTATGTGGAAGAACAAGCATGTCGTATCGAAATGCCCGGGCCGCCGTACCTCGCCAAGCAGGAGTGCTGTGAGCAGCTTGCAAACATTCCGCAGCAGTGCCGATGCCAGGCGCTGCGCTACTTCATGGGGCCGAAGTCTCGCCCGGATCAGAGCGGCCTCATGGAACTCCCCGGATGCCCTAGGGAGGTGCAGATGAACTTCGTCCCAATACTCGTCACTCCGGGGTACTGCAACTTGACGACCGTTCACAACACCCCATACTGCCTCGGTATGGAGGAGTCTCAGTGGAGCTAGAGACAATTCTCTCGCTCATGAATAAATAAGCATGTTGCGACCATACATGTGTGACATGCATATATACATATAGGACGAGCTCCGCGCGCTCATCATGTGTGGTGCTATCTGCTATATATATGGATAATAAGAATAAAGGGAATCATTTTCGCTTCTTAAAAA

WASI Bu-1

GCACGAGGCTCACGTGCAGCGCGGATCCGCCGCCGGTGCACGACACGGACGGCAACGAGCTGCGCGCCGACGCGAACTACTACGTCCTCCCGGCCAACCGCGCCCACGGCGGGGGGCTCACGATGGCGCCGGGCCACGGGCGCCGCTGCCCGCTGTTCGTCTCGCAGGAGGCCGACGGGCAGCGCGACGGCTTACCCGTGCGCATCGCCCCGCACGGCGGCGCGCCGTCCGACAAGATCATTCGGCTGTCGACCGACGTCCGCATCTCCTTCCGCGCCTACACGACATGCGTGCAGTCCACCGAGTGGCACATCGACAGCGAGCTGGTGTCGGGCCGCCGGCACGTGATCACCGGCCCGGTCAGGGACCCCAGCCCGAGCGGCAGGGAGAACGCCTTCCGCATCGAGAAGTACAGCGGCGCGGAGGTGCACGAGTACAAGCTCATGGCGTGCGGGGACTCGTGCCAGGACCTCGGCGTGTTCAGGGACCTCAAGGGCGGCGCATGGTTCTTGGGCGCCACCGAGCCATACCATGTCGTCGTGTTCAAGAAGGCGCCGCCCGCCTGAGGTGCATGCGGCGTGATGGCGCGCGCCCGTAGCGTGCGTGTGTGCATCTGAATAAGGCGGGCAGAAACAGTGTGCTGTTTTTGTCTCTCTTAGAGAATAAGTGTGGTGCTGTGCTGTCATCACTGCGTAGGCGGTTTCGTGAAACGTGAGAAACTTTTTTCAAAAAAAAAAAAAAAAAA

WASI Bu-2

TCGGCACGAGGCCCAAGCACGCCTTAGCTTGCAGTCTCGCTTGTCCTCGAGCACACACTAGCAGAGGTTTCAGCCATGAGTAGCCGCCGTGTTGGACTCCTCTTCATCTCCCTTCTGGCCATCGCCCTCTCGTGCAGCGCGGATCCGCCGCCGGTGCACGACACGGACGGCAACGAACTGCGCGCCGACGCGAACTACTACGTCCTCCCGGCCAACCGCGCCCATGGCGGGGGGCTCACGATGGCGCCCGGCCACGGGCGCCGCTGCCCGCTCTTCGTCTCGCAGGAGGCCGACGGGCAGCGCGACGGCTTGCCCGTGCGCATCGCCCCGCACGGCGGCGGCGCGCCGTCCGACAAGATAATTCGGCTGTCGACCGACGTCCGCATCTCCTTCCGCGCCTACACGACGTGCGTGCAGTCCACCGAGTGGCACATCGACAGCGAGCTGGTGTCCGGCCGCCGGCACGTGATCACCGGCCCGGTCAGGGACCCGAGCCCGAGCGGCAGGGAGAACGCCTTCCGCATCGAGAAGTACAGCTGGCGCGGAGGTGCACGAGTACAAGCTCATGGCATGCGGGGACTCGTGCCAGGACCTCGGCGTGTTCAGGGACCTCAAGGGCGGCGCGTGGTTCCTGGGCGCCACTGAGCCGTACCATGTCGTCGTGTTCAAGAAGGCGCCGCCCGCCTGATCGAGGTCCATGCGGCGTGATGGCGCGCTCCCGTAGCGTGCATGTGTGCATCT

CMx Bu-1

ACGAGCTGAGAATGGCGTTCAAGCACCAGCTCATCCTCTCCACCGCCATCCTGCTCGCCGTACTTGCCGCGGCGTCGGCCAGCTTCAGGGAACAATGCGTTCCAGGGCGGGAGATCACATACGAGTCGCTTAACGCCTGCGCCGAGTACGCGGTCAGACAAACATGCGGCTACTACCTCTCCGCCGAGAGGGAGAAGAGGCGGTGCTGCGACGAGCTGTCCAAGGTCCCGAAGTTCTGCCGGTGCGAGGTGCTGCACATCCTCATGGATGGGAGGGTGACTAAGGAGGGTGTGGTTAAGGGCAGCCTCCTCCAGGAGGACATGTCCAGATGCAAAAAGTTGACGAGGGAGTTCATCGCGGGCATCGTCGGGCGGGAGGAGTGCAACTTAGAGACTGTCTTAGGGCCGTACCACTACTGCCCGACTGAATATCCTGAAGTGGTTGTGTAACTTTATAAATAGCTCGAGCGTGAATAAGCTTGTTGCTCCATCGATGGACGAATCGTATATGTGTATCTATGCGCATGTGCGAAAAATATGGCAACAAAGAGCATCCGAGAGGATAATCTTAATCTTCAAAAAAAAAAAAAAAAAA

CMx Bu-2

ACGAGAGTTGAGAATGCCGTTCAAGCACCAGCTCCTCCTCTCGACCGCCGTCCTGCTCGCCGCACTTGCCGCGGGGTCGGCCAGCTTCAGGGACCGATGCGTTCCAGGGCGGGAGATCACATACGAGTCGCTTAACGCCTGCCGCGAGTACGCGGTCAGACAAACATGCGGCTACTACCTCTCCGCCGAGAGGCAGAAGAGGCGGTGCTGCGACGAGCTGTCCAAGGTCCCGGAGCTGTGCCGGTGCGAGGTGCTGCGCATCCTCATGGACGGGAGAGTGACTAAGGAGGGCGTGGTCAAGGGCAGCCTCCTCCAGGACATGTCCGGATGCAAAAAGCTGACGAGGGAGTTCATCGCGGGCATCGTCGGCCGGGAGGAGTGCAACTTGGAGACCGTCTTCGGGCGGTACCATTACTGCCCGTCCGAATATCTTGGACCTGAAGTGGTCGTGTAACTTTATAAATAGCTCGAGCGTGAATAAGCCTGTTGCTCGATCCATGGACGAATCGCATATGTGTATCCATGCGCATGTGCAAAAAATATGGCAACAAAGAGCATCCGAGAGGATGATGTTAGTCTTTAAAAAAAAAAAAAAAAAAAA

CMx Bu-3

GGCACGAGGAAAAGGAGGAGTTGAGAATGGCGTTCAAGCACCAGCTCATCCTCTCGACCGCCATCCTGCTCGCCGTACTTGCCGCGGCGTCGGCCAGCTTCAGGGACCGATGCGTCCCAGGGCGGGAGATCCCATACGAGTCGCTTAACGCCTGCCGCGAGTACGCGGTCAGACAAACATGCGGCTACTACCTCTCCGCCGAGAGGCTGAAGAGGCAGTGCTGCGACGAACTGTCCAAGGTCCCGGAGTTGTGCCGGTGCGAGGTGCTGCACATCCTCATGGATGGGAGAGTGACTAAGGAGGGTGTGGTTAAGGGCAGCCTCCTCAAGGACATGTCCGGATGCAAAAAGTTGACGAGGGAGTTCATCGCGGGCATCGTCGGGCGGGAGGAGTGCAACTTAGAGACTGTCTTCGGGCGGTACCATTATTGCCCGACCCAATATCCTGAAGTGGTCGTGTAACTTTATAAATAGCTCGAGCGTGAATAAGCTTGTTGCTCGATCGATGGACTAATCGCATATGTGTATCTATGCGCATGTGCGGAAAATATGGCAACAAAGAGCATCCGAGAGGATGATGTTTATCCAAGCATGCACTTTTTCCGCTTGCAACGACATGTGCAGCAGGATGGCGCGGNGACGCCAACATTTCACGTTTTGCACCCGCCCTGGCCAGTGCTGTTGTTGGTTTACATAGTTTGACCGGACACAACCGTTTTGGTTACTTGTGCGATGTAATCCTCCGGATGA

WTI Bu-1

CTCTCGGCTCTAGTCCTGCTCTCCATCCTCGCCGCCGCGGTAGCCACCATCGCCTGCAGGCCAGGGGTGGGCATCCCGCCCAAGCCACTCCCAAGCTGCCGCGCGTACGTGGTCCAGAAGACCTGCAAAGACACCCAACAGACCACTCCGGGAAAAGTGGCCTCCAAGGATCCGTGCTGTCGGGAGCTGGAAGCCGTCTCGGAAGAATGCCGATGCACCGCGATGGAGGACTTCATGCAAGGGATGCTCCGCTTGGAGGGCGTTCCTGAGGGGTGCACTAGAAAGGACCTGTGGGAGTTCACCTTGTCTCTCGTCAAGCCGGAGCTGTGTAACCTGAAGACCATCACCGGCGGGCCGTACTGCGGCCTCCCTCCTAGCAACGATGCTCGTCTTGCCGACAGTGTACAAGATGTGTAATTAAGCATGTGACTGATGGTGCATGATGAATAAGCATGTAAATTGCGCATGAAAAAAAAAAAAAAAAAAAA

WCI Bu-1

TTCGGCACGAGGACCAGAGAATATAAAACCAACCAACGTCCATCAATGGCGTCGTGCAGCCAGCACCTCCTCTCAGCCGTCGCCATCTTCTCCGTCCTGGCCGCCGCCGCCACCGCCACCAGTATGTACACCTGCTACGAAGGGGTGGGCCTCCCCGTGGACCCGCTCCAGGGCTGCCACTACTACGTGACCTCGCAGACCTGCGGCTTCGTGCCGCTGCTCCCAATTGAGGTGATGAAGGACCGGTGCTGCCGGGAGCTGGCCGCCATCTCGTCCAACTGCCGCTGCGAGGGGCTGCGCGTCTTCATCGACCGGGCGTTCCCTCCCAGCCAAAGCCAAGGCGGTGGCCCCCCGCAGCCGCCGCTGGCACCTAGGTGCCCGACGGAGGTGAAGAGGGACTTCGCCAGAACGCTCGCCCTGCCGGGGCAGTGCAACCTGCCGACCATCCATGGCGGCCCCTACTGCGTCTTCCCATGAACGCTAGCTTGTTCATGAAAACATTGTGTGAATGAATAAGCTAGCAGCATGTCACATCCGTGGATGGGTGATATGGTTACATGCATAAGTGCGCAATAAAGAGAATAATCTTCCGAA
