# Supplementary material for: The spectrum of low molecular weight alpha-amylase/protease inhibitor genes expressed in the US bread wheat cultivar Butte 86
Source: BMC Res Notes. 2011 Jul 20;4:242. doi: 10.1186/1756-0500-4-242 (PMC3154163; doi:10.1186/1756-0500-4-242)
Supplement: Additional file 3 — Amino acid sequences of alpha-amylase/protease inhibitors deduced from consensus sequences of Butte 86 contigs. [file 1756-0500-4-242-S3.DOC]

Additional file 3. Amino acid sequences of alpha-amylase/protease inhibitors deduced from consensus sequences of Butte 86 contigs.

WMAI Bu-1

MWMKTVFWGLLVFMLVATTMAVEYGARSHNSGPWSWCDPATGYKVSALTGCRAMVKLQCVGSQVPEAVLRDCCQQLADINNEWCRCGDLSSMLRSVYQELGVREGKEVLPGCRKEVMKLTAASVPEVCKVPIPNPSGDRAGVCYWAAYPDV

WMAI Bu-2

MLMKTVFWGLLLFMLVATTMAVEYGARSHNSGPWSWCDPATGYKVSALTGCRAMVKLQCVGSQVPEAVLRDCCQQLADINNEWCRCGDLSSMLRSVYQELGVREGKEVLPGCRKEVMKLTAASVPEVCKVPIPNPSGDGAGVCYWAAYPDV

WDAI-Bu 1

MSMKTVFSVLLLCMLVATPIAAEYDAWSVNSGPWMCYPGQAFQVPALPACRPLLRLQCNGSQVPEAVLRDCCQQLAHISEWCRCGALYSMLDSMYKEHGAQEGQAGTGAFPRCRREVVKLTAASITAVCRLPIVVDASGDGAYVCKDVAAYPDA

WDAI Bu-2

MSMKTMFSVLLLCMLVATPIAAEYDAWSGNSGPWMCYPGQAFQVPALPACRPLLRLQCNGSQVPEAVLRDCCQQLAHISEWCRCGALYSMLDSMYKEHGAQEGQAGTGAFPRCRREVVKLTAASITAVCRLPIVVDASGDGAYVCKDVAAYPDA

WDAI Bu-3

MSMKTVFSVLLLCMLVATPIAAEYDAWSVNSGPWMCYPGYAFKVPALPGCRPVLKLQCNGSQVPEAVLRDCCQQLADISEWCRCGALYSMLDSMYKEHGVQEGQAGTGAFPSCRREVVKLTAASITAVCKLPIVIDASGDGAYVCKGVAAYPDA

WDAI Bu-4

MKIVFSVLLLCMLVATPIASEYGAWSYNSGPWMCYPGQAFQVPALPGCRPLLKLQCNGSQVPEAVLRDCCQQLADISEWCRCGALYSMLDSMYKEHGVSEGQAGTGAFPSCRREVVKLTAASITAVCRLPIVVDASGDGAYVCKDVAAYPDA

WTAI-CM1 Bu-1

MASKSSISPLLLATVLVSVFAAATATGPYCYAGMGLPINPLEGCREYVAQQTCGISISGSAVSTEPGNTPRDRCCKELYDASQHCRCEAVRYFIGRRSDPNSSVLKDLPGCPREPQRDFAKVLVTPGHCNVMTVHNAPYCLGLDI

WTAI-CM2 Bu-1

MASKSSITHLLLAAVLVSVFAAAAATGPYCYPGMGLPSNPLEGCREYVAQQTCGVGIVGSPVSTEPGNTPRDRCCKELYDASQHCRCEAVRYFIGRTSDPNSGVLKDLPGCPREPQRDFAKVLVTPGHCNVMTVHNTPYCLGLDI

WTAI-CM3 Bu-1

MACKSSCSLLLLAAVLLSVLAAASASGSCVPGVAFRTNLLPHCRDYVLQQTCGTFTPGSKLPEWMTSASIYSPGKPYLAKLYCCQELAEISQQCRCEALRYFIALPVPSQPVDPRSGNVGESGLIDLPGCPREMQWDFVRLLVAPGQCNLATIHNVRYCPAVEQPLWI

WTAI-CM3 Bu-2

MACKSSCNLLLLAAVLLSVVAAASASGSCVPGVAFRTDLLPHCRDYVLQQTCGTFTPGSKLPEWMTSASIFSPMKPYLAKLYCCQELAEIPQQCRCEALRYFIALPVPSQPVDPRSGNVGESGLIDLPGCPRQMQWDFVRLLVAPGQCNLATIHNVRYCPAVEQLLWI

WTAI-CM16 Bu-1

MASKSNCVLLLAAVLVSIFAAVAAIGNEDCTPWMSTLITPLPSCRDYVEQQACRIETPGSPYLAKQQCCGELANIPQQCRCQALRYFMGPKSRPDQSGLMELPGCPREVQMDFVRILVTPGYCNLTTVHNTPYCLAMEESQWS

WTAI-CM17 Bu-1

MASKSNYNLLFAALLVFIFAAVAAVGNEDCTPWTSTLITPLPSCRNYVEEQACRIEMPGPPYLAKQECCEQLANIPQQCRCQALRYFMGPKSRPDQSGLMELPGCPREVQMNFVPILVTPGYCNLTTVHNTPYCLGMEESQWS

WASI Bu-1

DPPPVHDTDGNELRADANYYVLPANRAHGGGLTMAPGHGRRCPLFVSQEADGQRDGLPVRIAPHGGAPSDKIIRLSTDVRISFRAYTTCVQSTEWHIDSELVSGRRHVITGPVRDPSPSGRENAFRIEKYSGAEVHEYKLMACGDSCQDLGVFRDLKGGAWFLGATEPYHVVVFKKAPPA

WASI Bu-2

MSSRRVGLLFISLLAIALSCSADPPPVHDTDGNELRADANYYVLPANRAHGGGLTMAPGHGRRCPLFVSQEADGQRDGLPVRIAPHGGGAPSDKIIRLSTDVRISFRAYTTCVQSTEWHIDSELVSGRRHVITGPVRDPSPSGRENAFRIEKYSGAEVHEYKLMACGDSCQDLGVFRDLRGGAWFLGATEPYHVVVFKKAPPA

CMx Bu-1

MAFKHQLILSTAILLAVLAAASASFREQCVPGREITYESLNACAEYAVRQTCGYYLSAEREKRRCCDELSKVPKFCRCEVLHILMDGRVTKEGVVKGSLLQEDMSRCKKLTREFIAGIVGREECNLETVLGPYHYCPTEYPEVVV

CMx Bu-2

MPFKHQLLLSTAVLLAALAAGSASFRDRCVPGREITYESLNACREYAVRQTCGYYLSAERQKRRCCDELSKVPELCRCEVLRILMDGRVTKEGVVKGSLLQDMSGCKKLTREFIAGIVGREECNLETVFGRYHYCPSEYLGPEVVV

CMx Bu-3

MAFKHQLILSTAILLAVLAAASASFRDRCVPGREIPYESLNACREYAVRQTCGYYLSAERLKRQCCDELSKVPELCRCEVLHILMDGRVTKEGVVKGSLLKDMSGCKKLTREFIAGIVGREECNLETVFGRYHYCPTQYPEVVV

WTI Bu-1

LSALVLLSILAAAVATIACRPGVGIPPKPLPSCRAYVVQKTCKDTQQTTPGKVASKDPCCRELEAVSEECRCTAMEDFMQGMLRLEGVPEGCTRKDLWEFTLSLVKPELCNLKTIGGPYCGLPPSNDARLADSVQDV

WCI Bu-1

MASCSQHLLSAVAIFSVLAAAATATSMYTCYEGVGLPVDPLQGCHYYVTSQTCGFVPLLPIEVMKDRCCRELAAISSNCRCEGLRVFIDRAFPPSQSQGGGPPQPPLAPRCPTEVKRDFARTLALPGQCNLPTIHGGPYCVFP
